# Tourette Syndrome and Klippel-Feil Anomaly in a Child with Chromosome 22q11 Duplication

**DOI:** 10.1155/2009/361518

**Published:** 2009-12-22

**Authors:** Raymond A. Clarke, Zhi Ming Fang, Ashish D. Diwan, Donald L. Gilbert

**Affiliations:** ^1^St George Clinical School, Faculty of Medicine, St George Hospital, University of NSW, Kogarah, NSW 2217, Australia; ^2^Movement Disorders Clinics, Division of Pediatric Neurology, Cincinnati Children's Hospital Medical Center, ML # 11006 - Neurology, 3333 Burnet Avenue, Cincinnati, OH 45229-3039, USA

## Abstract

This is the first case description of the association of Klippel-Feil Syndrome (KFS), Tourette Syndrome (TS), Motor Stereotypies, and Obsessive Compulsive Behavior, with chromosome 22q11.2 Duplication Syndrome (22q11DupS). Neuropsychiatric symptoms in persons with 22q11.2 deletion, including obsessive compulsiveness, anxiety, hyperactivity, and one prior case report of TS, have been attributed to low copy number effects on Catechol-O-Methyltransferase (COMT). However, the present unique case of 22q11DupS and TS suggests a more complex relationship, either for low- or high-COMT activity, or for other genes at this locus.

## 1. Introduction

Klippel-Feil Syndrome (KFS) involves a congenital fusion of vertebrae in the cervical spine [1]. Tourette Syndrome (TS) is a heritable neuropsychiatric disorder characterized by persistent, childhood-onset tics that fluctuate in intensity, migrate in anatomic location, and are performed in response to sensory urges [2]. Motor stereotypies usually have younger onset, are much more stable phenomenologically, and occur involuntarily with excitement or other stimulation [3]. Longstanding clinical observations that high doses of amphetamines can induce stereotypies and tics [4], while dopamine receptor blocking agents inhibit them [5], support a hyperdopaminergic neurotransmission or receptor hypersensitivity model for these symptoms, although the dopamine/tic relationship is likely much more complex [6]. 

Large genetic association and linkage studies to date have failed to implicate genes for dopamine synthesis or metabolism as causes of TS, but clinical presentations of rare genetic diseases involving dopamine pathways are supportive, as in a recently reported case of TS in a girl with chromosome 22q11 deletion [7]. This region contains Catechol-O-Methyltransferase (COMT), which degrades dopamine, norepinephrine, and epinephrine. Low-COMT gene copy number has been associated with obsessive compulsive and hyperactive psychopathology in velocardiofacial syndrome [8], and lesser-COMT activity theoretically could increase catecholamines and hyperkinetic movements.

We report clinical and genetic characterization of the first case of 22q11DupS and TS, KFS, and stereotypies, suggesting that both low and high copy numbers of 22q11 may generate hyperkinetic and obsessive compulsive symptoms.

## 2. Case Presentation

The patient, a Caucasian male diagnosed with KFS, was referred at age of 9 years for evaluation of repetitive movements. He presented with motor stereotypy in the 1st year of life which persists to the present: a patterned movement involving the flapping of both hands, and sometimes body and leg stiffening is occurring daily, with excitement. From age 6 to his present age, 12 years, he has had a series of mild tics including eye blinking, nose twitching, leg and toe pointing, and repetitive coughing and sniffing, consistent with a diagnosis of TS. He also has obsessive compulsive behaviour (OCB) and anxiety, with excessive rumination, but no Attention Deficit Hyperactivity Disorder (ADHD). He has seen a psychologist for dysthymia and adjustment but takes no neurologic or psychiatric medications. At school, academic performance is average to above. Past Medical History includes full-term birth, at 3.1 kg. Development includes walking by 16 months and speaking normally by 2 years. Surgical history includes a Woodward Procedure for Sprengel's Deformity of the scapula and Cervical Spine Decompression and Stabilization. Review of systems includes parasomnias and migraines. Family history is negative for tics, OCB, or other neurologic or psychiatric disorders.

General examination revealed a barrel-chested appearance, a short neck with low posterior hairline, abnormal scapulae, and webbing of toes. At age of 12 years, weight was 36.6 kg (75%ile), height was 126.4 cm (<5%ile), body mass index was 34 (>95%ile), and head circumference was 50.5 cm. Palate and cardiac exams were normal. Neurologic exam was notable for normal language and gregarious social interactions. Occasional tics and the stereotypy were observed. Cranial nerve exam was normal. Distal strength was diminished in both hands and thumb abductors. Reflexes were 2+ and symmetric. Fine movements of the hands were clumsy and slow. Sensation was diminished to light touch in the hands. Current clinical ratings showed Yale Global Tic Severity Scale (YGTSS) total tic score of 6 (mild), Child Yale-Brown Obsessive Compulsive Scale (CY-BOCS) score of 16 (mild/moderate), and the DuPaul ADHD Rating Scale (ADHDRS) score of 17 (subthreshold).

**Figure 1:**
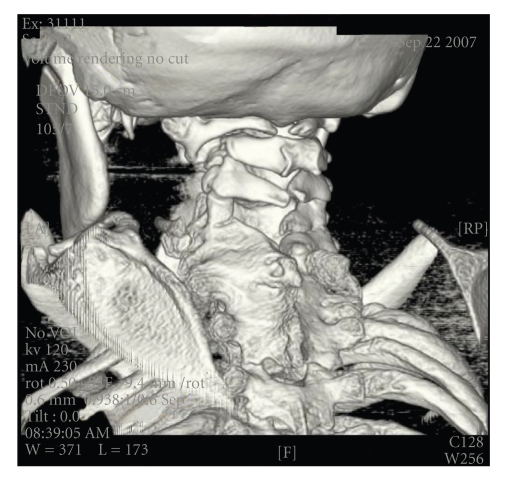
Computed Tomography of the C Spine, with 3D reconstruction, showing Klippel-Feil anomaly comprising of the fusion of multiple cervical and upper thoracic vertebrae.

Imaging studies include CT scans that revealed fusion of cervical and thoracic vertebrae (Figure 1), scoliosis, hypoplastic thumbs, and several digits of the feet and hands.

Due to the complex phenotype, we evaluated the patient's DNA using comparative genomic hybridization (CGH) analysis (BeadChip technology with a Single Nucleotide Polymorphism based array; Illumina HD Human610-quad BeadChip platform). This revealed a ~3 Mb hemizygous microduplication of chromosome 22q11.2, the same region as the classical 22q11 microdeletion syndrome [9], as well as the 22q11.2 duplication with a less well-characterized phenotype [10]. This region contains genes coding for approximately 20 proteins expressed in brain and/or bone.

Followup negative testing of the parents demonstrated that this duplication was de novo.

**Figure 2:**
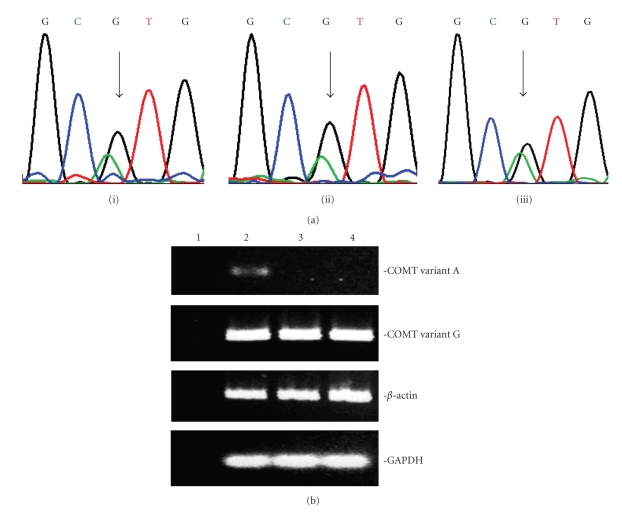
(a) Nucleotide sequence chromatograms across the COMT G675A haplotype using (i) patient DNA, (ii) patient cDNA, (iii) mRNA/cDNA from a normal control heterozygote. (b) Expression of the COMT G675A variants. COMT amplicons from semiquantitative RT-PCR were visualized on an agarose gel after 33 cycles using *β*_2_-actin and GAPDH (housekeeping genes) as the quantitation control. Lane 1—no cDNA control, lane 2—patient, lane 3 & 4—normal control individuals that were homozygous for the 675G allele.

To further characterize this boy's COMT genotype, we generated a lymphocyte cell line. DNA nucleotide sequence analysis indicated that the patient was heterozygous for the G675A COMT haplotype (Figure 2(a)i) and that the chromatogram peak height for the 675G variant was consistently two times higher than that of the 675A variant (Figure 2(a)i). This suggests that the 675G (higher-expressing) allele [11] was duplicated. We then compared mRNA/cDNA sequence for the patient and a normal individual. The G : A ratio for the normal control heterozygote was slightly >1 (Figure 2(a)iii) consistent with the instability of the 675A mRNA transcript [11]. In contrast, the G : A ratio for the patient was >2 (Figure 2(a)ii) which again suggested that the patient has a higher than normal level of expression of the COMT gene.

To directly compare COMT gene expression levels, we optimized a proven two-step, high-temperature, semi-quantitative RTPCR protocol (denature at 94°C for 30 seconds followed by extension at 71°C for 40 seconds for 33 cycles) [12] using primers specific for either the 675G or 675A: the forward 675G primer (COMT-ValF 5′-ATGGTGGATTTCGCTGGCGT-3′) and the 675A primer (COMT-MetF 5′-ATGGTGGATTTCGCTGGCAT-3′) were positioned within exon 4 of the COMT gene, and the reverse primer (COMT-R 5′-CTTCCGCAGCAGGCCACATT-3′) was positioned in exon 5. Only the patient (Figure 2(b), lane 2) expressed the 675A allele and both the patient and two normal controls (lanes 3 and 4) had comparable levels of expression of the 675G allele, as evident from the agarose gel electrophoresis of the amplicons.

To assess possible clinical implications of this finding, cerebrospinal fluid was obtained via lumbar puncture for assessment of neurotransmitter levels. Routine cells (1 white, 1 red blood cell), protein, and glucose were normal. Levels of CSF neurotransmitter metabolites were obtained through HPLC testing commercially (Medical Neurogenetics, Atlanta, Georgia). The level of 3-O-methyldopa, a metabolite of L DOPA via COMT, was normal at 19 nmol/L (normal <100). The level of Homovanillic acid, a final common pathway metabolite of dopamine and norepinephrine via COMT, was normal at 279 nmol/L (167–563).

## 3. Discussion

We describe a boy with KFS, TS, stereotypy, and obsessive compulsive symptoms in association with de novo 22q11DupS with normal parental. This is the first report of TS associated with 22q11DupS. The incidence of TS in the general population is sufficiently high that this association could be incidental. However, a 22q11.2 deletion has been reported in association with tics and TS [7], and as neither parent had a personal or family history of these neuropsychiatric symptoms, our results suggest that the 22q11DupS may confer risk for the TS phenotype. 

Hemizygous microdeletion at 22q11.2 (22q11DS) is the most common microdeletion syndrome, usually involving homologous recombination of the same ∼3 Mb region duplicated in our patient [13]. Within the 22q11.2 duplication region, candidate genes implicated in central nervous system structure or function include *CLTCL1, RTN4R, SNAP29, PRODH, GSCL*, *UFDIL, ES2*, and COMT. The COMT gene (MIM no. 116790) has particular interest because of its role in monoamine degradation and because a common 675G > A (Val158Met) substitution results in the dysregulation of the dopaminergic system. The 675A allele of the COMT haplotype codes for the ^158^ *Met* variant of this membrane-bound enzyme that is expressed at lower levels in the brain compared to the alternate ^158^ *Val* variant [14]. A recent investigation of 126 normal healthy Caucasian subjects indicated the COMT low-expression/low-activity A allele forms part of a more expansive COMT gene haplotype “G-A-A” that appears to associate with inefficient prefrontal working-memory response [15]. This same COMT haplotype (G-A-A) also appears to be more prevalent in 22q11DS patients with OCD and ADHD [16].

Both low copy number in 22q11DS and reduced COMT expression due to the presence of a COMT 675A allele [14] have been associated with neuropsychiatric symptoms, including anxiety and Obsessive Compulsive Symptoms [17, 18]. Cerebrospinal fluid neurotransmitter and metabolite levels were not altered in our patient. Although this finding might have had treatment implications, it was not surprising. A recent report showed no alterations in these studies in psychiatric patients with COMT gene polymorphisms [19]. Another study found CSF dopamine changes in Restless Legs Syndrome, but mainly in the presence of more severe symptoms [20]; our patient has milder motor tic symptoms. Finally, it is worth pointing out that studies have questioned the role for low COMT activity in psychiatric disorders [21, 22]. Our patient was heterozygous for the G675A COMT haplotype with duplication or the 675G, higher-expressing COMT allele. As such, the 22q11.2 duplication for this patient calls into question a unique role of low COMT activity in the etiopathogenesis of OCD or TS.

Outside the brain, the 22q11DS and 22q11DupS manifest considerable phenotypic overlap, including congenital heart defects, palatal defects and abnormal facies, micrognathia, short stature, dysplastic ears and hearing defects, down-slanting palpebral fissures, urogenital anomalies, absent thymus, T cell deficiency, anomalies of the hands and feet, and scoliosis, suggesting diverse effects of both low and high copy numbers [23]. Mouse studies indicate that many of the physical anomalies associated with copy number variation at 22q11.2 are attributable to *Tbx1* dosage [24]. Mutations in the *GDF6* gene, which is expressed in the developing intervertebral space, are causative for the KF2 class of KFS [25]. *Tbx1* is likewise expressed in the developing intervertebral space [25, 26]. The 22q11DS has shown prior association with synostosis in the appendages, scoliosis, and vertebral irregularities [9]; however, this is the first report of vertebral fusion in association with duplication or deletion at 22q11.2. We propose *Tbx1* as a candidate gene for broad spectrum KFS where it presents in association with Sprengle's shoulder, heart, and palatal and/or urogenital anomalies.

## 4. Conclusion

This study is the first to report an association between TS and 22q11DupS, thereby broadening the link between copy number variation at 22q11.2 and the development of TS and comorbid neurobehavioral disorders including OCB. Further research should delineate the basis for linkage between one or more genes within the 22q11.2 ∼3 Mb critical recombination region and neuropsychiatric disorders.
